# Individual Differences in Narcissism: From Personality to Psychopathology

**DOI:** 10.3390/bs16101846

**Published:** 2026-10-09

**Authors:** Elias Tsakanikos, Mark Hardaker

**Affiliations:** 1 Department of Psychology, University of Crete, 74150 Rethymno, Greece; 2 Department of Psychosis Studies, Institute of Psychiatry, Psychology & Neuroscience, King’s College London, London SE5 8AF, UK

## 1. Narcissus Revisited in Contemporary Research

In Ovid’s telling, Narcissus perishes at the pond, undone by his own reflection. In Pausanias’ lesser-known version, he gazes at the water to recover the likeness of a beloved twin sister who has died. Same youth, same pond, two rather different stories. One of vanity, the other of loss. The scholarly literature has arrived at a similar juncture. Narcissism, a trait reflecting egocentric exceptionalism and social selfishness, has splintered into forms, such as grandiose versus vulnerable, agentic versus communal, admirative versus rivalrous (Back et al., 2013; Krizan & Herlache, 2018), that predict unique outcomes and, on occasion, opposite ones (Miller et al., 2017). It has also acquired uneasy neighbours (psychopathy, Machiavellianism; Paulhus & Williams, 2002) and an ambiguous relation to. Where personality ends and psychopathology begins is, if anything, less obvious than it was (Pincus & Lukowitsky, 2010; Sedikides, 2021).

This Special Issue was conceived to take stock of these developments. The nine contributions span cross-sectional and multi-study surveys, a daily diary design, a round-robin design in a naturalistic team setting, network analysis, implicit reaction-time paradigms, and a randomised experiment. Although they differ considerably in method and emphasis, they converge on a common conclusion: the form of narcissism under examination determines, to a substantial degree, the pattern of results obtained. In what follows, the contributions are organised under four themes, (measurement and self-esteem, grandiosity and vulnerability, narcissism within the Dark Triad, and narcissism in social context) before we consider the directions in which the field might proceed.

## 2. Overview of the Contributions

### 2.1. Narcissism and the Mirror: Measurement and Self-Esteem

Narcissism and self-esteem have been separated conceptually, but the instruments used to assess narcissism have not always followed suit. Altmann and Roth (Contribution 6) compared several widely used narcissism scales in a sample of 337 participants and examined the extent to which each overlaps with global self-esteem. The overlap varied considerably between instruments. Vulnerable measures showed the greatest overlap, and their correlations with the Big Five, empathy, and aggression changed most once self-esteem was controlled; grandiose and antagonistic measures were generally more robust, although differences emerged among these as well. The implication is clear. Narcissism measures cannot be treated as interchangeable, and researchers who wish to isolate narcissistic disposition from self-regard will need to select instruments deliberately, control for self-esteem, or both.

Hardaker and Tsakanikos (Contribution 9) approached the same relation experimentally. Self-esteem was modelled bifactorially (self-competence and self-derogation), grandiose narcissism was assessed with a three-factor model of the Narcissistic Personality Inventory (Power, Exhibitionism, Special Person), and 180 undergraduates were randomly assigned to a self-affirmation or control condition before completing a masked priming lexical decision task. Self-affirmation increased self-competence and reduced self-derogation. More notably, it attenuated the association between Special Person narcissism and the processing of negative status-related cues. These findings are consistent with the mask model, offer one account of the conflicting correlational evidence on narcissism and self-esteem, and provide, in addition, an experimental validation of a bifactorial model of self-esteem. The evidence is clear but preliminary therefore further investigations are needed. 

### 2.2. Two Roads to Perfection: Grandiosity and Vulnerability

Perfectionism provides a natural testing ground for the grandiose–vulnerable distinction. Schneider, Kornberger, Aßmuth, and Mokros (Contribution 3) examined 210 adults and found that grandiose narcissism predicted other-oriented perfectionism (the demand that others be flawless), whereas vulnerable narcissism predicted socially prescribed perfectionism (the perception that others demand flawlessness of oneself). The vulnerable pathway was largely mediated by social aspects of fear of failure, in particular the fear that important others will lose interest. Only vulnerable narcissism predicted faster implicit failure avoidance in a lexical approach–avoidance task. The two forms thus appear to be underpinned by distinct motivational orientations and to follow distinct routes to psychopathology.

### 2.3. Narcissism in Dark Company

Narcissism is conventionally grouped with psychopathy and Machiavellianism, and three contributions ask what binds the three traits. Mahadevan and Jordan (Contribution 4), across two studies (*n* = 591), located the answer in status motivation. Individuals high in Dark Triad traits strongly desire status, perceive themselves as having attained it, and behave so as to acquire more; they neither seek nor believe they have attained inclusion. This pattern held for the agentic and antagonistic dimensions but not for the impulsive ones, which offers a useful indication of where the ‘dark core’ resides. French, Loas, and Hein (Contribution 5) turned attention inward. In 492 French-speaking adults, Dark Triad scores were associated with depression, alexithymia, and anhedonia even after adjustment for depression severity. Notably, these associations were confined to women, in whom Machiavellianism and psychopathy in particular were linked to emotional distress. McIlvenna, Truhan, and Papageorgiou (Contribution 8) adopted a network approach (*n* = 350). Narcissistic extraversion was protective against depression and narcissistic neuroticism was a risk factor for it, but both operated through Machiavellian agency, a bridge node that Dark Triad research has tended to overlook. The Dark Triad, on this evidence, is dark but not merely aversive.

### 2.4. Narcissism in Social Context

Most of what is known about narcissism derives from questionnaires completed in isolation. Three contributions examine it in social settings. Zeigler-Hill, Toma, Thomas, and Besser (Contribution 1) demonstrated, across three studies, that extraverted and antagonistic narcissism were indirectly associated with anti-immigrant attitudes through a competitive social worldview and, in turn, right-wing authoritarianism and social dominance orientation; neurotic narcissism showed the opposite association. Hart, Carnelley, Vowels, and Thomas (Contribution 2) followed 196 partnered adults through a daily diary of perceived partner phubbing. Individuals higher in rivalry reported lower self-esteem and more negative mood on ordinary days and, when phubbed, greater resentment, conflict, and retaliation, the latter motivated by a desire to get even and to seek approval elsewhere. Individuals higher in admiration fared better on most indices, although they too reported more conflict on high-phubbing days. Bush-Evans, Hart, Cisek, Satchell, and Sedikides (Contribution 7) assigned 101 participants to 23 escape-room teams and obtained round-robin ratings. Those high in admirative narcissism were perceived as confident but overestimated their likeability. Those high in rivalrous narcissism were perceived as aggressive and lazy. Teams characterised by high rivalry showed reduced cohesion and, in consequence, poorer performance, whereas admiration had no team-level effects. Narcissism, it appears, is more readily observed in a shared task than in a self-report scale.

## 3. Concluding Remarks and Future Directions

Four themes run through the collection. First, the specific type or dimension of narcissism as a polyhedric construct (Sedikides, 2021) matters. Admiration and rivalry, grandiosity and vulnerability, extraversion and neuroticism diverged in study after study, and a global narcissism score would have concealed much of what was found. Second, antagonism accounts for most of the interpersonal cost of narcissism in fractured teams, namely relational conflict, status contests, and prejudice. Third, narcissism is not solely an externalising and directly observable phenomenon: fear of failure, alexithymia, anhedonia, and depression recur throughout the issue, and the distance from personality to psychopathology is shorter than the ‘dark’ label implies. Fourth, the relation between narcissism and self-esteem is a matter of refinement of measurement and, apparently, of experimental testing and validation of such mesures. Some long-standing disagreements in the field may be resolved once both are taken into account.

Several directions for future research follow. The field would benefit from a greater proportion of experimental relative to correlational designs; from behavioural observation in teams, couples, and clinical settings alongside self-report; from intensive-longitudinal designs that capture the day-to-day fluctuation of narcissistic states rather than their trait averages; from systematic attention to sex and culture as moderators; and from clinical samples that extend these findings across the threshold into Narcissistic Personality Disorder. Interventions, of which self-affirmation is one candidate, merit systematic evaluation. Narcissus, after all, was never afforded a second look at the pond. We are grateful to the authors, the reviewers, and the editorial staff of Behavioral Sciences for making this collection possible, and we hope that it offers the field a clearer reflection of its subject.

## Data Availability

No new data were created or analyzed in this study. Data sharing is not applicable to this article.
